# 

**Published:** 1914-07-18

**Authors:** 


### CONTENTS.

PAGE

**A Hospital Equerry for the King** ... ... 425
**Over-Insurance** ... ... ... ... ... ... 426
**Hospital and Institutional News** ... ... ... 427
Perth Royal Infirmary—A Munificent Commemoration of Their Majesties' Glasgow Visit—The Dundee Children's Hospital—The Massachusetts General Hospital—The Royal Devon and Exeter Hospital—A Manchester Babies' Hospital—New Accident Hospital at Manchester—Details of the Accommodation—The British Hospital for Mothers—Incorporation of Great Ormond Street Hospital—Hospital Extension at Willesden—A Curiosity of a Hospital—Two Bournemouth Institutions—The Manchester Radium Fund—A Question of Residence—The New Officers of the Royal College of Surgeons—A Health Census in Bradford—The Birmingham Brick League—The Control of Nursing Homes—The Nurses' Registration Bill Again—The Feeding of Hospital Nurses—Norwich Footballers and their Hospital—A Novel Method of Raising Funds—The Unalotted Panel Fund in London—Problems Arising out of School Inspection—Long-Service Medals for Nurses—This Week's Drug Market.
**Toxæmia in the Acute Specific Fevers...** ... 433
**The Theory of Fowler's Posture** ... ... 434
**The Charity Organisation Society and the Treatment of Tuberculosis in London** ... 435
**The School Doctor** ... ... ... ... ... 436
The Relation of the School Doctor to the Private Practitioner.

[See over.]

### CONTENTS—(*continued*).

PAGE

**Reports on Hospitals of the United Kingdom**— 437
By SIR HENRY BURDETT, K.C.B., K.C.V.O.
Caterham Cottage Hospital.
The Epsom and Ewell Cottage Hospital (Second Notice).

**Institutional Organisation in America** ... 439
II. A Lesson from the Massachusetts General Hospital, Boston.

**Hospital Statistics** ... ... ... ... ... 441

I. Methods of Keeping the Records of Patients.

**Round the World** ... ... ... ... ... 443

Fellow-Workers and their Doings.

**Hospital Architecture and Construction** ... 444
The Nurses' Home at the Massachusetts General Hospital.

**Maternity and Mothering** ... ... ... ... 445
The Influence of the Insurance Act upon the Practice of Midwifery.

PAGE

**The Editor's Letter-Box** ... ... ... ... 447
Bath Workhouse Infirmary — Children's Country Holidays Fund.

**Enteritis at Southwark Infirmary** ... ... 447

**Buildings Contemplated** ... ... ... ... 448

**Medical Appointments** ... ... ... ... 448

**From the Nurses' Point of View**... ... ... 449
A Reply to the Criticisms of a Patient.

**The Profession of Medicine** ... ... ... 450
Medical Defence.
The Shortage in the Naval Medical Service.

**The Institutional Worker** ... ... (Suppl.) 1
Destructors for the Disposal of Hospital Refuse.
A Perfect Hospital Door-hinge.
Questions for July.
Questions Invited.
Employment and Training.
The Book Market.

